# Value of Pharmacy-Based Influenza Surveillance — Ontario, Canada, 2009

**Published:** 2013-05-24

**Authors:** Jeffery J. Aramini, Pia K. Muchaal, Frank Pollari

**Affiliations:** Intelligent Health Solutions, Inc., Ontario; Public Health Agency of Canada

As part of ongoing efforts by the Public Health Agency of Canada (PHAC) to enhance disease surveillance, a retrospective epidemiologic study was undertaken to investigate the usefulness for influenza surveillance of data on changes in the volume of prescriptions for antiviral medications. The weekly numbers of dispensed prescriptions for the antiviral medications oseltamivir and zanamivir, as a proportion of all dispensed prescriptions, were compared with the numbers of confirmed laboratory reports of influenza A(H1N1) at the local health authority level in Ontario, Canada, during the second wave of the outbreak of pandemic influenza A(H1N1) in 2009. Qualitative and quantitative analyses demonstrated that antiviral prescription dispensing dates were a reasonable proxy for influenza A(H1N1) onset dates at the local health authority level. This report describes the results of those analyses, which indicated that 1) antiviral prescription proportions increased in advance of laboratory reports of influenza and 2) antiviral dispensing data can be available in near real-time. These findings suggest that pharmacy prescription data can provide timely intelligence to help characterize local influenza activity.

The value of influenza surveillance depends in part on the timeliness of the generated information. Traditional methods of influenza surveillance, including FluWatch (Canada’s national surveillance system), rely on the collection and aggregation of laboratory results and clinical observations from physicians and public health authorities. Typical for infectious diseases, it can take several days to weeks from symptom onset to data being collected, aggregated, and analyzed (1,2). Pharmacy-based surveillance uses near real-time dispensing data of pharmaceuticals (prescription and over-the-counter drugs) as a proxy for illness in the population. The potential for pharmacy-based surveillance to detect changes in community illness levels earlier than traditional laboratory-based surveillance methods is premised on the fact that the public will routinely seek over-the-counter medications to relieve or alleviate common symptoms of illness, and physicians often will prescribe medications before receiving laboratory confirmation (3,4). Retrospective disease outbreak studies have demonstrated increases in pharmaceutical sales before the recognition of increased illness frequency using traditional public health surveillance methods (5,6).

In this study, the proportion of dispensed prescription medications that were oseltamivir or zanamivir were compared each week with the number of confirmed laboratory reports of influenza A(H1N1) at the local health authority level. Prescription medication data (from 2009) were provided to PHAC by Rx Canada, Inc., and included individual-level prescription data from approximately 75% of Ontario’s community pharmacies (n = 1,202). Each prescription identified the drug, date of dispensing, and the patient’s sex and age. Laboratory reports of influenza A(H1N1) (from 2009) were provided to PHAC by the Ontario Ministry of Health and Long-Term Care.* Each laboratory report provided one of three dates: illness onset date, date specimen was submitted to the laboratory, and date laboratory results were reported to a public health authority. When case onset date was not available, it was estimated based on the mean time differences between date types. Each laboratory report included patient age and sex, and was linked to one of Ontario’s 36 local health authorities.

The relationship between antiviral prescriptions and influenza A(H1N1) laboratory-confirmed cases was investigated using a Poisson regression model. Potential correlation at the local health authority level was accommodated using a generalized estimating equation approach to determine parameter estimates. Weekly antiviral prescriptions dispensed (antivirals per 10,000 other prescriptions) were compared with weekly influenza A(H1N1) case counts. Prescription proportions were used (rather than absolute prescription counts) in an effort to adjust for a number of potential factors, including day-of-the week, holidays, and regional variation in physician prescribing patterns. Lagged weekly influenza A(H1N1) case counts were used to investigate the potential time-lag between influenza A(H1N1) symptom onset dates and antiviral prescription dispensing dates.

During July 1–December 31, 2009, information was available on approximately 43,000 Ontario oseltamivir and zanamivir prescriptions. Patient age and sex were available for 82% of antiviral prescriptions: mean age was 34 years, median age was 33 years, and 57% of patients were female. During this period, information was available on approximately 7,300 Ontario influenza A(H1N1) laboratory confirmations: mean age of patients was 24 years; median age was 18 years, and 47% were female patients. Symptom onset date was available for 56% of the cases, laboratory specimen date for 32% of the cases, and laboratory reporting date for 12% of the cases. The average time difference from mean (median) onset date to mean (median) specimen date was 6 (5) days, and from mean (median) specimen date to mean (median) reporting date was 6 (8) days.

Very little if any lag was observed between the influenza A(H1N1) case onset trend line and the antiviral prescription trend line (Figures 1 and 2). Poisson regression analysis demonstrated a statistically significant relationship between weekly influenza A(H1N1) case counts and antiviral prescriptions at the local health authority level (p<0.001). Statistical significance was greatest when influenza A(H1N1) cases counts were not lagged by time. Analysis results were similar when only the 56% of cases with known onset date were considered.

What is already known on this topic?

Traditional methods of influenza surveillance rely on the collection and aggregation of laboratory results and clinical observations from physicians and public health authorities. It can take several days to weeks from symptom onset to data being collected, aggregated, analyzed, and reported.

What is added by this report?

Changes in the ratio of prescriptions for two drugs prescribed for the prophylaxis and treatment of influenza to all other prescriptions coincided with the second wave of the influenza pandemic in Ontario, Canada, during July 1–December 31, 2009. Prescriptions tracked dates of symptom onset ahead of dates of positive influenza laboratory reports at the local health authority level.

What are the implications for public health practice?

Infectious disease mitigation strategies are most effective when implemented early. Real-time surveillance of pharmacy data might be more useful than laboratory data for guiding early implementation of these strategies.

## Editorial Note

The findings in this report demonstrate that during the second wave of the influenza A(H1N1) epidemic in 2009 in Ontario, antiviral prescription dispensing mirrored influenza A(H1N1) onset activity at the local level with no appreciable lag time. These results suggest that pharmacy-based surveillance can provide a mechanism to monitor and detect influenza-like activity regardless of whether the underlying pathogen is laboratory confirmed. This might be especially important if the pathogen is not routinely tested for.

The time lag between symptom onset and laboratory reporting to public health officials of a known pathogen can be substantial (2). Even during the second wave of the influenza A(H1N1) outbreak, when public health authorities in Ontario were prepared, an average time lag from symptom onset to reporting of an influenza A(H1N1) confirmation to public health authorities was estimated to be 12 days. If the cause of an influenza-like illness is unknown or not routinely tested for (e.g., a novel coronavirus), the gains achieved in timeliness with pharmacy-based surveillance might be much greater.

The reporting of positive influenza laboratory results in a community likely contributed to increased physician prescribing of antivirals. However, given an estimated 12-day lag time from symptom onset to laboratory reporting to public health authorities, publicized influenza laboratory confirmations likely did not influence prescription patterns during the early phases of increased community activity.

The findings in this report are subject to at least three limitations. First, although analysis results were similar regardless of whether the 44% of cases with estimated onset dates were considered, the validity of estimating onset dates based on specimen or reported date cannot be assessed. Second, the proportion of prescriptions administered for prophylaxis versus treatment is not known, neither is the effect this might have had on the temporal association between onset dates and prescription dispensing dates. Finally, this study focused on one event, the 2009 influenza A(H1N1) pandemic. Additional investigation involving more years of data and more geographic locations are required before any findings can be generalized.

Although laboratory-based surveillance remains a cornerstone of influenza surveillance, the need for more timely surveillance data has never been greater. With the routine and daily movement of persons between communities, an infectious disease can rapidly spread around the world in a matter of days. In addition, much has been learned about how infectious diseases like influenza spread and what methods can and should be used to help minimize spread and potential impacts. Successful results of most mitigation strategies (e.g., cough etiquette, hand washing, staying home when sick, and vaccination reminders) are best achieved if implemented in the community as early as possible.

The contribution of pharmacy-based surveillance to an overall influenza surveillance strategy primarily depends on the timeliness of the pharmacy data. In Canada, as in most industrialized nations, the pharmacy industry maintains sophisticated information systems to manage drug inventory and client data. An ongoing PHAC real-time pharmacy-based surveillance project demonstrates that the collection, aggregation, and analysis of near real-time prescription data from thousands of community pharmacies from across Canada is readily achievable.

## Figures and Tables

**FIGURE 1:**
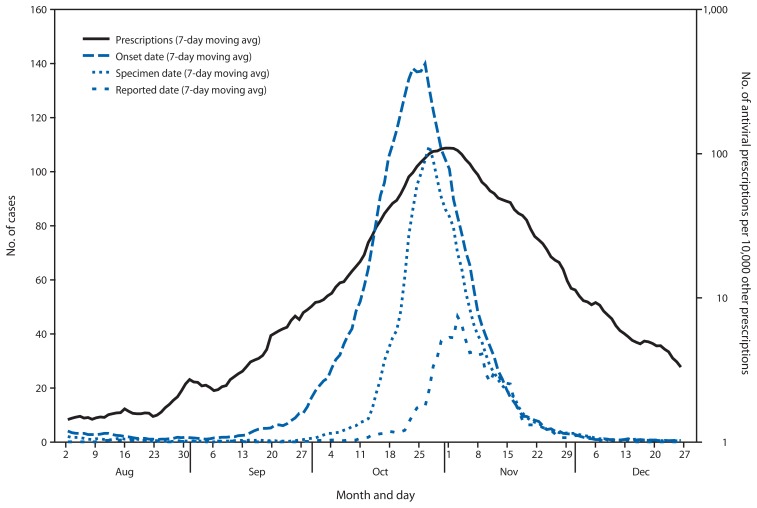
Seven-day moving average number of reported influenza A(H1N1) cases and number of antiviral prescriptions per 10,000 other prescriptions — Ontario, Canada, August–December 2009

**FIGURE 2:**
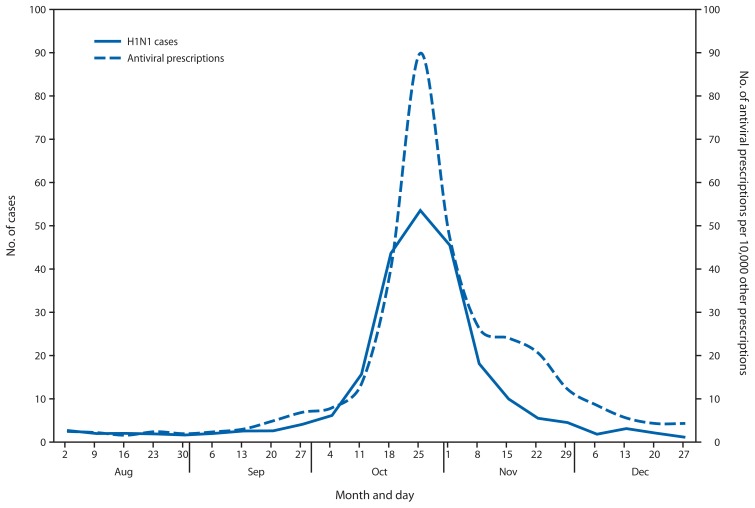
Average weekly number of influenza A(H1N1) cases and number of antiviral prescriptions per 10,000 other prescriptions reported at the local health authority level — Ontario, Canada, August–December 2009

## Footnotes

* Extracted by Public Health Ontario from the Integrated Public Health Information System database on March 21, 2011.
